# Extensive alternative splicing triggered by mitonuclear mismatch in naturally introgressed Rhinolophus bats

**DOI:** 10.1002/ece3.7966

**Published:** 2021-07-28

**Authors:** Wenli Chen, Xiuguang Mao

**Affiliations:** ^1^ School of Ecological and Environmental Sciences East China Normal University Shanghai China; ^2^ Institute of Eco‐Chongming (IEC) East China Normal University Shanghai China

**Keywords:** gene expression, horseshoe bats, introgressive hybridization, mitonuclear interaction, transcriptome

## Abstract

Mitochondrial function needs strong interactions of mitochondrial and nuclear (mitonuclear) genomes, which can be disrupted by mitonuclear mismatch due to mitochondrial DNA (mtDNA) introgression between two formerly isolated populations or taxa. This mitonuclear disruption may cause severe cellular stress in mismatched individuals. Gene expression changes and alternative splicing (AS) are two important transcriptional regulations to respond to environmental or cellular stresses. We previously identified a naturally introgressed population in the intermediate horseshoe bat (*Rhinolophus affinis*). Individuals from this population belong to *R. a*. *himalayanus* and share almost identical nuclear genetic background; however, some of them had mtDNA from another subspecies (*R. a*. *macrurus*). With this unique natural system, we examined gene expression changes in six tissues between five mitonuclear mismatched and five matched individuals. A small number of differentially expressed genes (DEGs) were identified, and functional enrichment analysis revealed that most DEGs were related to immune response although some may be involved in response to oxidative stress. In contrast, we identified extensive AS events and alternatively spliced genes (ASGs) between mismatched and matched individuals. Functional enrichment analysis revealed that multiple ASGs were directly or indirectly associated with energy production in mitochondria which is vital for survival of organism. To our knowledge, this is the first study to examine the role of AS in responding to cellular stress caused by mitonuclear mismatch in natural populations. Our results suggest that AS may play a more important role than gene expression regulation in responding to severe environmental or cellular stresses.

## Graphical abstract

It describes a study on the role of alternative splicing in response to cellular stress caused by mitonuclear mismatch in naturally introgressed populations, usually resulted from differential introgression of mitochondrial DNA. Our current study suggests that alternative splicing sometimes may play a more important role than gene expression regulation in responding to severe environmental or cellular stresses in nature.

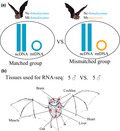

## 1 INTRODUCTION

Mitochondria play vital roles in the survival and fitness of the organism (Hill, 2015; Wallace, 2010). Its proper function requires strong interactions between mitochondrial and nuclear (mitonuclear) genomes (Hill, 2015; Rand et al., 2004). In order to maintain compatibility of these two genomes, natural selection promotes mitonuclear co‐evolution (Barreto et al., 2018; Bar‐Yaacov et al., 2012; Levin et al., 2014; Sloan et al., 2017) and leads to the co‐adaptation of mitonuclear genotypes within each isolated population (Hill et al., 2019; Sloan et al., 2018). However, when different isolated populations having respective co‐adapted mitonuclear genotypes come into secondary contact, subsequent differential introgression between them disrupts mitonuclear interactions in introgressed individuals (Burton & Barreto, 2012; Burton et al., 2013).

Disruption of mitonuclear interactions, also called mitonuclear mismatch, can cause inefficiency of the oxidative phosphorylation (OXPHOS) pathway (e.g., decrease of adenosine triphosphate (ATP) production, Ellison & Burton, 2006) and lead to a higher level of reactive oxygen species (ROS) production in mitochondria (Gusdon et al., 2007; Rand et al., 2018). These will result in increased oxidative damage at the cellular level (e.g., lipids, proteins, and DNA, Balaban et al., 2005; Barreto & Burton, 2013; Vives‐Bauza et al., 2006). Therefore, individuals with mismatched mitonuclear genotypes tend to have a lower survival rate and fitness than ones with matched genotypes (Barreto & Burton, 2013; Latorre‐Pellicer et al., 2016; Rank et al., 2020; Vaught et al., 2020). In order to cope with the environmental stresses, such as the cellular stress resulting from mitonuclear mismatch (Ballard & Towarnicki, 2020), cells and organisms need to respond quickly and efficiently.

Changes in gene expression have been widely implicated in the rapid adaptation to variable environmental stresses (Fraser, 2013; Hodgins‐Davis & Townsend, 2009; Kenkel & Matz, 2016; Lasky et al., 2014; López‐Maury et al., 2008; Rivera et al., 2021), phenotypic variations (Hodgins‐Davis & Townsend, 2009; Kaern et al., 2005; Mank, 2017), and adaptive radiations (Barrier et al., 2001; El Taher et al., 2021; Whittall et al., 2006). Alternative splicing (AS), a mechanism of creating multiple isoforms from a single gene, offers another route for organisms to respond to environmental stresses rapidly and efficiently (Kijewska et al., 2018; Smith et al., 2018) and has also been associated with phenotypic variations in eukaryotes (Bush et al., 2017; Grantham & Brisson, 2018). Compared with gene expression regulation, AS has been proposed to play a more important role in facilitating rapid adaptive divergence within short timescales (Jacobs & Elmer, 2021; Singh et al., 2017). Several previous studies have demonstrated significant changes in gene expression between mitonuclear mismatched and matched individuals (Flight et al., 2011; Healy et al., 2017; Mossman et al., 2016, 2017, 2019; Santiago et al., 2021). However, as far as we know, no studies have been conducted to examine the role of AS in response to the cellular stress caused by mitonuclear mismatch in natural populations.

Here, we fill this knowledge gap by studying bats which are the only mammals capable of flight. Because flight requires huge energy demands, energy production in mitochondria should be more efficient in bats than in other nonflight and similar‐sized mammals. Consistent with this difference in energy demand between bats and nonflight mammals, adaptive evolution of genes involved in energy metabolism has been associated with the origin of flight in bats (Shen et al., 2010). Therefore, bats are a good system to study the effects of inefficiency of energy production in mitochondria on the survival or fitness of organisms. In this study, we focus on a horseshoe bat (*Rhinolophus affinis*) which includes three subspecies in China. Two of them are from the mainland of China (*R. a*. *himalayanus* and *R. a*. *macrurus*) and a third is from Hainan Island. These subspecies have diverged recently, less than one million years ago (Mao et al., 2010). Our previous study on this species identified a hybrid zone between the two mainland subspecies in eastern region of China (Mao et al., 2010, 2013, 2014). We found frequent and extensive introgression of mitochondrial DNA (mtDNA) from *R. a*. *macrurus* to *R. a*. *himalayanus* with little or no nuclear introgression between them (Mao and Rossiter, 2020). This mtDNA introgression led to mitonuclear mismatch in some *himalayanus* individuals. Therefore, a *himalayanus* population may contain individuals with either mitonuclear mismatched or matched genotypes. We sampled one such population in our previous study (Ding et al., 2021). Using whole‐genome resequencing and RNA‐seq data, we showed that this *himalayanus* population included two groups of individuals with almost identical nuclear genetic backgrounds but divergent mtDNA due to introgression of mtDNA from *macrurus* to one group (Ding et al., 2021).

Previously, we used this unique system to investigate the effects of mitonuclear mismatch on nuclear gene expression based on RNA‐seq data from six adult tissues (Ding et al., 2021). Although our previous results demonstrated significant nuclear gene expression changes in mismatched individuals compared with matched ones, we identified a small number of differentially expressed genes (DEGs) in a majority of tissues (less than 10 in all six tissues except for muscle). Most of these DEGs were found to be related to immune response, and some might play important roles in protecting the cell against the oxidative damage (Ding et al., 2021). However, none was directly or indirectly involved in OXPHOS and related to energy production or other processes in mitochondria. This suggests that gene expression changes might be limited by functional constraints (see also Rogers et al., 2021) and therefore sometimes inefficient in responding to the cellular stresses generated within a short timescale.

In the current study, we aimed to examine the role of AS in responding to the cellular stress caused by mitonuclear mismatch. We quantified differences in AS between mitonuclear mismatched and matched individuals in each of the six tissues using the same RNA‐seq data from Ding et al. (2021). If AS plays a more important role in coping with the cellular or environmental stresses than gene expression regulation, as shown in previous studies (Jacobs & Elmer, 2021; Singh et al., 2017), we expect to find some alternatively spliced genes (ASGs) which are involved in energy production and other mitochondrial processes.

## 2 MATERIALS AND METHODS

### 2.1 Ethics statement

Our sampling and tissue collection procedures were approved by the National Animal Research Authority, East China Normal University (approval ID bf20190301).

### 2.2 Sampling and RNA‐seq data collection

**FIGURE 1:**
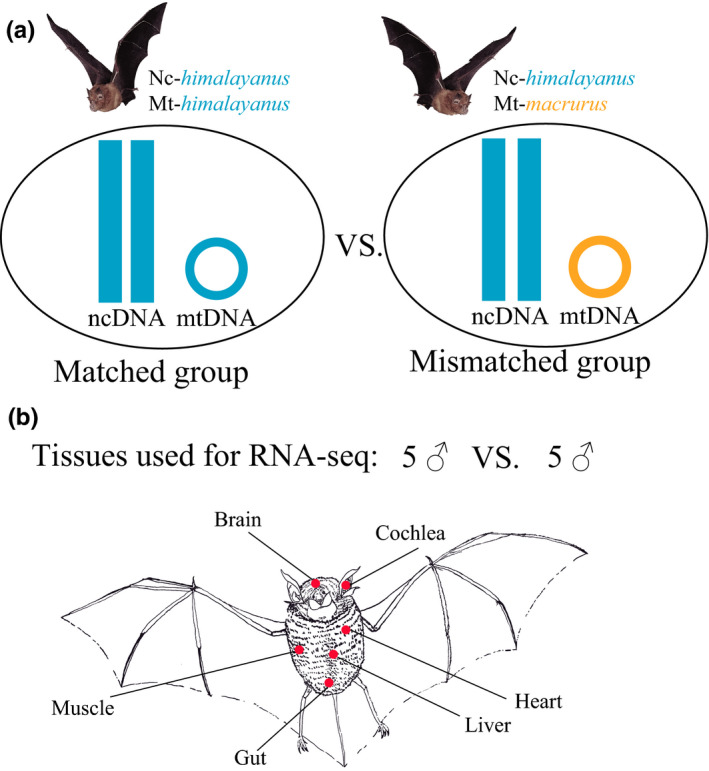
The study system used in this study (modified from Ding et al. (2021)). (a) Two groups were included, mitonuclear matched and mismatched groups. (b) Five male individuals were sampled in each group, and six tissues (muscle, heart, brain, liver, cochlea, and gut) were collected in each individual for RNA‐seq

Ten adult males from one population of *Rhinolophus affinis himalayanus*, collected in Anhui Province, China, in 2019, were used in this study (Figure 1 and Table S1). We previously confirmed that these 10 *himalayanus* individuals exhibited near‐identical nuclear genetic background using whole‐genome resequencing and RNA‐seq data (Ding et al., 2021). However, five of them showed divergent mitochondrial DNA (mtDNA) due to introgression of mtDNA from *R. a. macrurus* (Ding et al., 2021). Therefore, we divided these 10 *himalayanus* individuals into two groups: mitonuclear mismatched group and matched group. The former included five individuals with *macrurus* mtDNA (Nc‐*himalayanus*:Mt‐*macrurus*), and the latter included five individuals with *himalayanus* mtDNA (Nc‐*himalayanus*:Mt‐*himalayanus*) (Figure 1 and Table S1, see also Ding et al., 2021).

For each bat, six tissues were collected including muscle, heart, brain, liver, cochlear, and gut. For each tissue sample, library constructions were performed using Illumina's TruSeq mRNA Stranded Library Preparation Kit and sequenced with Illumina HiSeq X Ten (paired‐end 150 bp). RNA‐seq data for a total of 59 tissue samples (one from muscle was discarded due to the low quality library) were obtained from Ding et al. (2021) (BioProject accession no. PRJNA727985).

### 2.3 Raw reads trimming and mapping

Raw sequencing reads from each sample were trimmed with TRIMMOMATIC version 0.38 (Bolger et al., 2014) using a sliding window of 4 bp with minimum average PHRED quality score of 20 and minimum read length of 50 bp. Because rMATs, the program for alternative splicing analysis, requires all input reads to be of equal length, we trimmed reads to 140bp using TRIMMOMATIC and removed those with <140bp. Trimmed reads were mapped to the reference genome of *R. a. himalayanus* (unpublished data from Gang Li) using Hisat2 (Kim et al., 2015) with a minimum acceptable alignment score of −86. The resulting mRNA alignments were used in both alternative splicing and differential expression analysis.

### 2.4 Differential expression analysis

We used similar procedures to perform differential expression (DE) analysis as in Ding et al. (2021). Briefly, mapped reads in mRNA alignments above were quantified using featureCounts (Liao et al., 2014). We then removed the lowly expressed genes with a mean CPM (counts per million) less than one in each group. Read count matrices across samples were normalized in DESeq2 (Love et al., 2014). Five samples (one brain, one liver, two cochlear, and one gut) were identified as significant outliers using *PcaGrid* method (Croux et al., 2007) implemented in the *rrcov* R package with default parameters. It should be noted that one liver sample that was identified as an outlier in Ding et al. (2021) was not an outlier in this study and a cochlear sample that was identified as an outlier here was not in Ding et al. (2021). All outlier samples were excluded before further analysis. We then used DESeq2 to identify differentially expressed genes (DEGs) between the two *himalayanus* groups (mitonuclear mismatched and matched groups) in each tissue using *p* value <.05 after Benjamini and Hochberg adjustment for multiple tests (*p*_adj_ < .05, Benjamini & Hochberg, 1995).

### 2.5 Alternative splicing analysis

We used rMATs v4.1.0 (Shen et al., 2014) to detect the alternative splicing (AS) events and alternatively splicing genes (ASGs) between the two *himalayanus* groups in each tissue. To ensure comparisons of AS and DE differences in each tissue, the same number of samples was used in AS analysis as in the DE analysis above. The initial outputs from rMATs included the PSI value of each splicing event, indicating the proportion of isoform in mismatched group to isoform in matched group at each splice site. Following Rogers et al. (2021), we identified AS events using 0 < PSI < 1 in at least half of the samples in each group. To compare AS between groups, rMATs calculates the inclusion difference (ΔPSI, average PSI of mismatched group minus average PSI of matched group). ΔPSI ranges from 1 (the isoform is only expressed in mismatched group) to −1 (the isoform is only expressed in matched group). Significance of ΔPSI between the two groups was determined using a likelihood‐ratio test. To ensure using the same thresholds as in DE analysis above, we identified AS events between the two groups using the false discovery rate (FDR) <.05.

### 2.6 Functional enrichment analysis

We performed functional enrichment analysis using Metascape (Zhou et al., 2019, http://metascape.org), and five ontology categories were selected including gene ontology (GO) biological process, KEGG pathway, Reactome Gene Sets, WikiPathways, and Hallmark Gene Sets. All expressed genes in the six tissues (a total of 15,256 genes) were included as the background list. We used a *p* value of <.01 to determine significant terms which were grouped into clusters. *p*‐values are calculated with the accumulative hypergenometric distribution (Zar, 1999).

## 3 RESULTS

### 3.1 Effects of mitonuclear mismatch on nuclear gene expression

In order to use the same set of RNA‐seq reads in DE and AS analysis, we trimmed reads to 140bp. Although there is a small difference in the number of RNA‐seq reads used in this study compared with Ding et al. (2021) (see Table S2), we identified almost same sets of differentially expressed genes (DEGs) in each tissue as in Ding et al. (2021) if |log_2_ (fold change)| > 1 and *p*_adj_ < 0.05 were used (see Table S3 and Figure S1). Most DEGs identified in each tissue are shared between this study and Ding et al. (2021) except for liver (Figure S1). Specifically, five DEGs in liver identified in Ding et al. (2021) were not DEGs in this study. This difference may have resulted from different samples used in DE analysis because only one sample was identified as an outlier in this study, while two samples were outliers in Ding et al. (2021).

**FIGURE 2:**
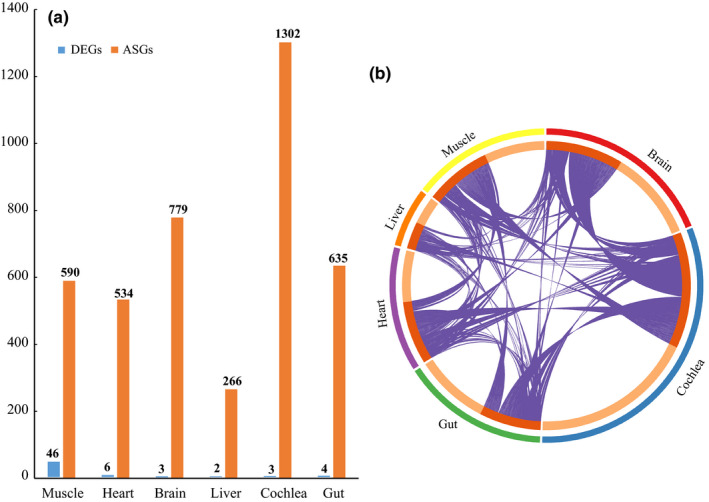
Differential expression and alternative splicing analysis between matched and mismatched groups across six tissues. (a) Bar plot showing the number of differentially expressed genes (DEGs) and alternatively spliced genes (ASGs) identified in each tissue. (b) Circos plot showing the overlapped ASGs across the six tissues

In order to make proper comparisons between DE and AS analysis, we only used *p*_adj_ < 0.05 to determine the DEGs in each tissue. Overall, similar to Ding et al. (2021), except for muscle in which 46 DEGs were identified, the remaining five tissues exhibited <10 DEGs (Figure 2a and Table S3) and no overlapped DEGs were found across the six tissues and between pairs of tissues. Similar to Ding et al. (2021), significantly enriched categories were only found in DEGs identified in muscle tissue and a majority of them are related to immune response (Figure S2 and Table S4). It is notable that one enriched category is associated with response to SARS‐CoV‐2 (WP4884: pathogenesis of SARS‐CoV‐2 mediated by nsp9‐nsp10 complex).

### 3.2 Effects of mitonuclear mismatch on alternative splicing

We quantified AS events between mitonuclear matched and mismatched groups across six tissues. Overall, we identified a large number of AS events and alternatively spliced genes (ASGs) with an average of 5.1% of expressed genes in each tissue (Table [Link], [Link] and [Link], [Link]; Figure 2a) and a total of 4,106 ASGs in all six tissues. We detected five different types of AS events in each tissue including skipped exons (SE), mutually exclusive exons (MXE), alternative 5′ and 3′ splice site (A5′SS and A3′SS), and retained intron (RI) (see detailed description of each type in Rogers et al., 2021). MXE and SE generally showed higher frequency than other types of AS events across tissues except in brain and cochlea (Table S5). Next, we compared patterns of tissue differences in AS. We found that most ASGs were specific to each tissue and none was shared across the six tissues (Figure 2b). However, shared ASGs were detected between pairs of tissues (Figure 2b). Among pairwise comparisons, brain and cochlea shared the largest number of ASGs.

**FIGURE 3:**
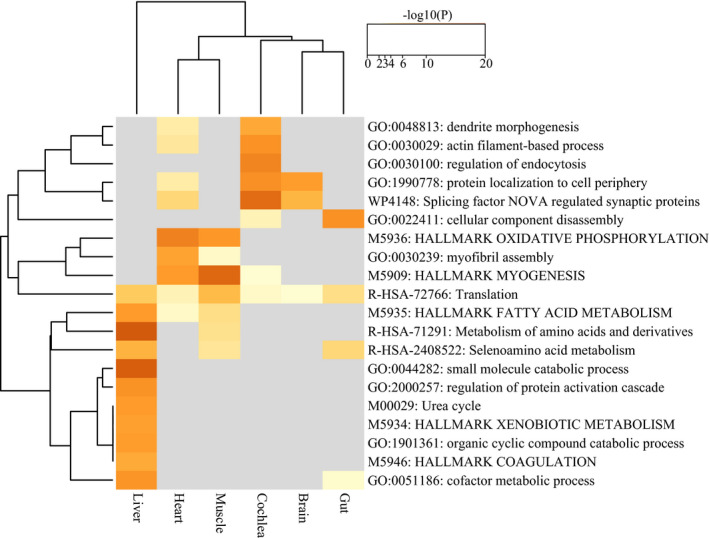
Heatmap showing the top 20 enriched ontology categories across alternatively spliced genes (ASGs) identified in all six tissues. Each category is colored by p‐values, and gray color represents a lack of significance. See Figure S4 for the 100 enriched categories across ASGs identified in all six tissues

In contrast to the case of DEGs above, functional enrichment analysis revealed a large number of significant categories enriched across ASGs identified in each tissue. Overall, heatmap of enriched categories across the six tissues revealed that ASGs in muscle and heart shared more categories than ASGs in cochlea, brain, and gut (Figure 3). In contrast, most enriched categories in ASGs of liver were specific to this tissue (Figure 3). Specifically, multiple ASGs in muscle are enriched in categories directly involved in energy production in mitochondria, such as OXPHOS or respiratory electron transport chain, including several GO terms (GO: 0033108; GO: 0055114; GO: 0010257; GO: 0032981; GO: 0022900), Hallmark Gene Sets (M5936), KEGG Pathway (hsa00190), Reactome Gene Sets (R‐HSA‐163200), and WikiPathways (WP4921; WP111; WP4324) (Figure S3; see details in Table S7). In addition, we also found several enriched categories associated with other mitochondrial processes, such as mitochondrion organization (GO: 0007005), mitochondrial translation (GO: 0032543), and mitophagy (R‐HSA‐5205647). Like the case in muscle, in heart we found several enriched categories involved in OXPHOS, including GO terms (GO: 0055114; GO: 0043462), Hallmark Gene Sets (M5936), KEGG Pathway (hsa00190), and WikiPathways (WP111) (Figure S3; see details in Table S7). We also found multiple shared categories between muscle and heart whose functions are associated with muscle contraction, myofibril assembly, and myogenesis. In brain, we found several categories related to energy production (e.g., GO: 0009435: NAD biosynthetic process). In addition, some ASGs in brain were found to be associated with response to stress (R‐HSA‐2559580: oxidative stress‐induced senescence; R‐HSA‐2262752: cellular responses to stress). In liver, a majority of enriched categories are related to metabolisms among which several are associated with energy production in mitochondria, such as NAD biosynthetic and metabolic process (GO: 0009435; GO: 0019674) and oxidoreduction coenzyme metabolic process (GO: 0006733). Although the number of enriched categories in cochlear was the largest among the six tissues, we did not find categories directly or indirectly involved in energy production. However, we identified several ones which are important for mitochondrial function, such as mitochondrion disassembly (GO: 0061726) and autophagy of mitochondrion (GO: 0000422). The above two categories (GO: 0061726 and GO: 0000422) are also observed in gut. In addition, multiple ASGs in gut are found to be associated with DNA damage response (GO: 0043518) and ROS pathway (M5938).

## 4 DISCUSSION

Mitonuclear mismatch due to mtDNA introgression between two formerly isolated populations or taxa may disrupt the proper mitonuclear interactions, leading to mitochondrial dysfunction in mismatched individuals (Barreto & Burton, 2013). Mitochondrial dysfunction usually results in a decreased rate of ATP production and thus an increased rate of ROS generation, which will cause severe cellular stress (Ballard & Towarnicki, 2020; Ellison & Burton, 2006). Gene expression changes and alternative splicing (AS), acting as important transcriptional regulations, have been shown to play essential roles for cells and organisms in responding to environmental or cellular stresses efficiently (Ibrahim et al., 2021; Lee et al., 2021).

We previously found a naturally introgressed population from *R. a. himalayanus* in which all ten individuals share almost identical nuclear genetic background, while some of them have the mitochondrial genome from another parapatric subspecies—*R. a*. *macrurus* (Ding et al., 2021). We used this unique system to examine the effects of mitonuclear mismatch on nuclear gene expression. This study confirms results of our previous study that mitonuclear mismatch could alter nuclear gene expression in multiple tissues (Ding et al., 2021), which have also been documented in other organisms (e.g., *Drosophila*, Mossman et al., 2016; Mossman et al., 2017; Mossman et al., 2019; killfish, Flight et al., 2011, Healy et al., 2017). Functional enrichment analysis revealed that a majority of differentially expressed genes (DEGs) between mismatched and matched individuals were related to immune response, suggesting that cellular immune surveillance could be enhanced in mismatched individuals in order to cope with the effects of mitonuclear mismatch. A recent study has also shown that mitochondrial dysfunction could trigger nuclear immune response (Tigano et al., 2021). However, a small number of DEGs were identified in mismatched individuals compared with matched ones, indicating modest effects of mitonuclear mismatch on nuclear gene expression (see also Healy et al., 2017). In addition, we did not identify DEGs directly or indirectly involved in energy production in mitochondria or other mitochondrial processes. These suggest that gene expression regulation might be sometimes inefficient in responding to the cellular stresses due to functional constraints (see also Rogers et al., 2021).

In contrast to gene expression regulation above, we identified extensive number of AS events and ASGs between mitonuclear mismatched and matched individuals. To our knowledge, this is the first study to assess the role of AS in response to the cellular stress caused by mitonuclear mismatch in natural populations. Consistent with our hypothesis that AS plays a more important role in coping with the cellular or environmental stresses than gene expression regulation, functional enrichment analysis revealed that multiple ASGs identified in muscle and heart were involved in OXPHOS and thus directly related to energy production in mitochondria. In addition, ASGs identified in remaining four tissues were enriched categories indirectly associated with energy production, including NAD biosynthetic process, oxidoreduction coenzyme metabolic process, mitochondrion disassembly, and autophagy of mitochondrion. In muscle, brain, and gut, we also found several ASGs whose function may be involved in the cellular response to stresses (e.g., oxidative stress and DNA damage). Thus, our study supports that AS can act as an efficient mechanism in responding to environmental or cellular stresses, which has been extensively implicated in both animals and plants (Grantham & Brisson, 2018; Ibrahim et al., 2021; Kijewska et al., 2018). More importantly, our current study further indicates that AS may play a more important role than gene expression regulation in coping with the severe cellular stress in nature (see also Singh et al., 2017; Jacobs & Elmer, 2021; Martín et al., 2021).

## CONFLICT OF INTEREST

The authors declare that they have no competing interests.

## AUTHOR CONTRIBUTION

**Wenli Chen:** Formal analysis (supporting); Writing‐review & editing (supporting). **Xiuguang Mao:** Conceptualization (supporting); Project administration (supporting); Writing‐original draft (supporting).

## Supporting information

Figure S1

Figure S2

Figure S3

Figure S4

Table S1

Table S2

Table S3

Table S4

Table S5

Table S6

Table S7

## Data Availability

RNA‐seq data for a total of 59 tissue samples were obtained from Ding et al. (2021) (BioProject accession no. PRJNA727985). All data generated or analyzed during this study are included in this published article and its supplementary information files.
