# Supplementary material for: Extensive alternative splicing triggered by mitonuclear mismatch in naturally introgressed Rhinolophus bats
Source: Ecol Evol. 2021 Jul 28;11(17):12003–10. doi: 10.1002/ece3.7966 (PMC8427577; doi:10.1002/ece3.7966)
Supplement: Supplementary file 1 — Figure S1 [file ECE3-11-12003-s002.pdf]

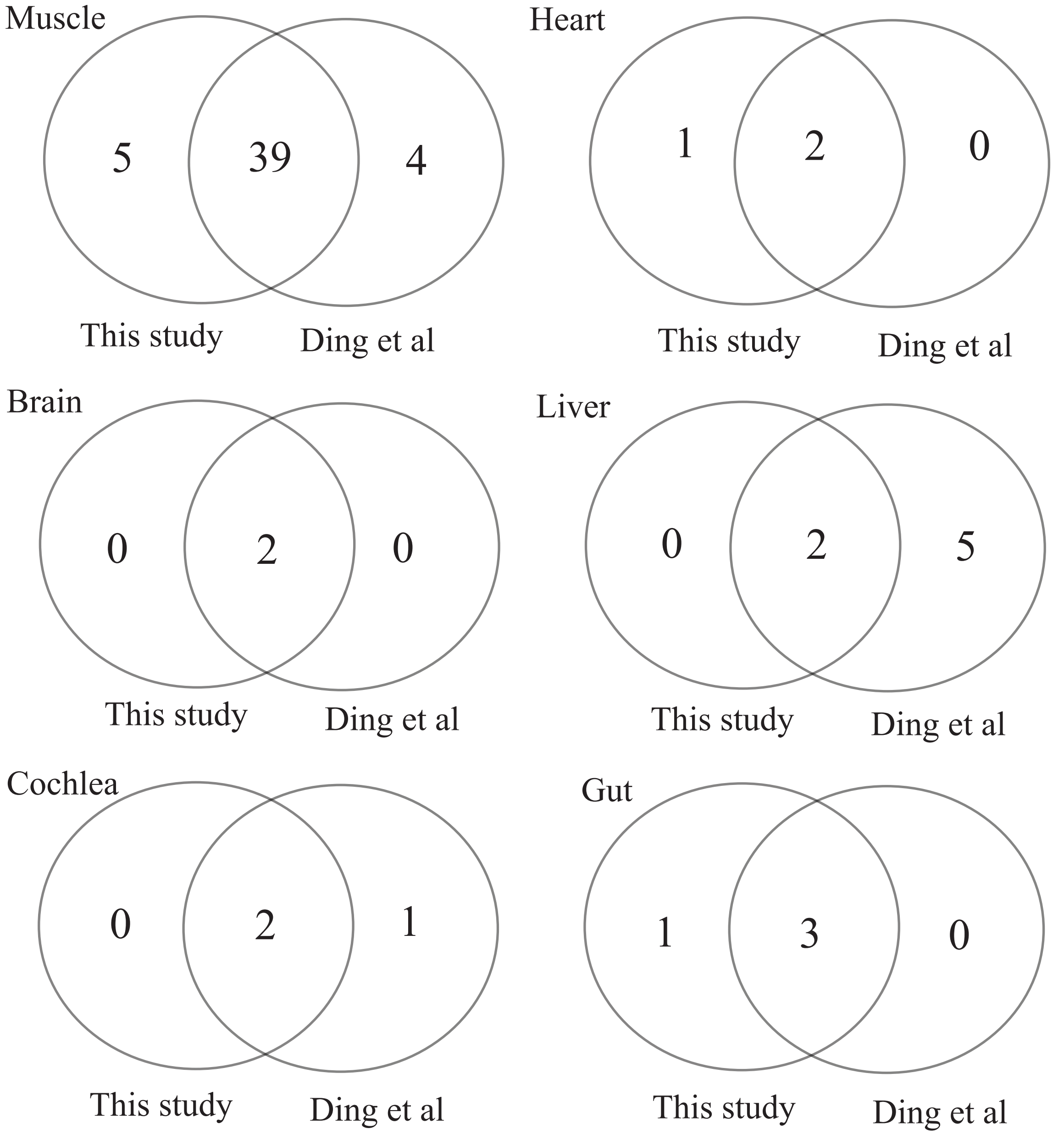
Muscle
5
39
4
This study
Ding et al
Heart
1
2
0
This study
Ding et al
Brain
0
2
0
This study
Ding et al
Liver
0
2
5
This study
Ding et al
Cochlea
0
2
1
This study
Ding et al
Gut
1
3
0
This study
Ding et al
