# Supplementary figures and images for: Extensive alternative splicing triggered by mitonuclear mismatch in naturally introgressed Rhinolophus bats
Source: Ecol Evol. 2021 Jul 28;11(17):12003–10. doi: 10.1002/ece3.7966 (PMC8427577; doi:10.1002/ece3.7966)

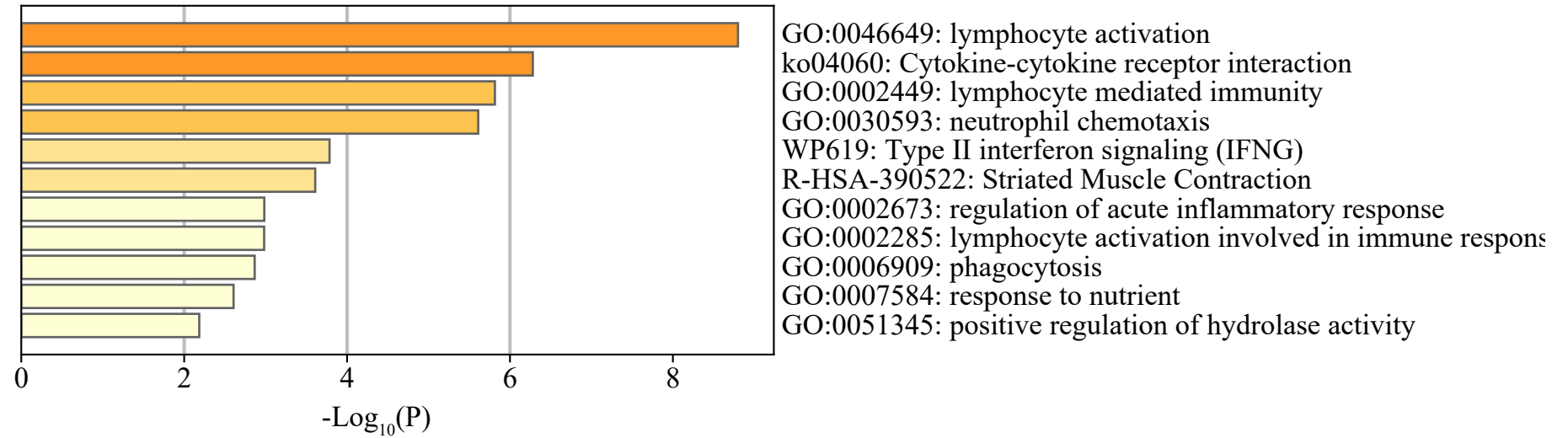

Supplement: Supplementary file 2 — Figure S2 [file ECE3-11-12003-s001.pdf]

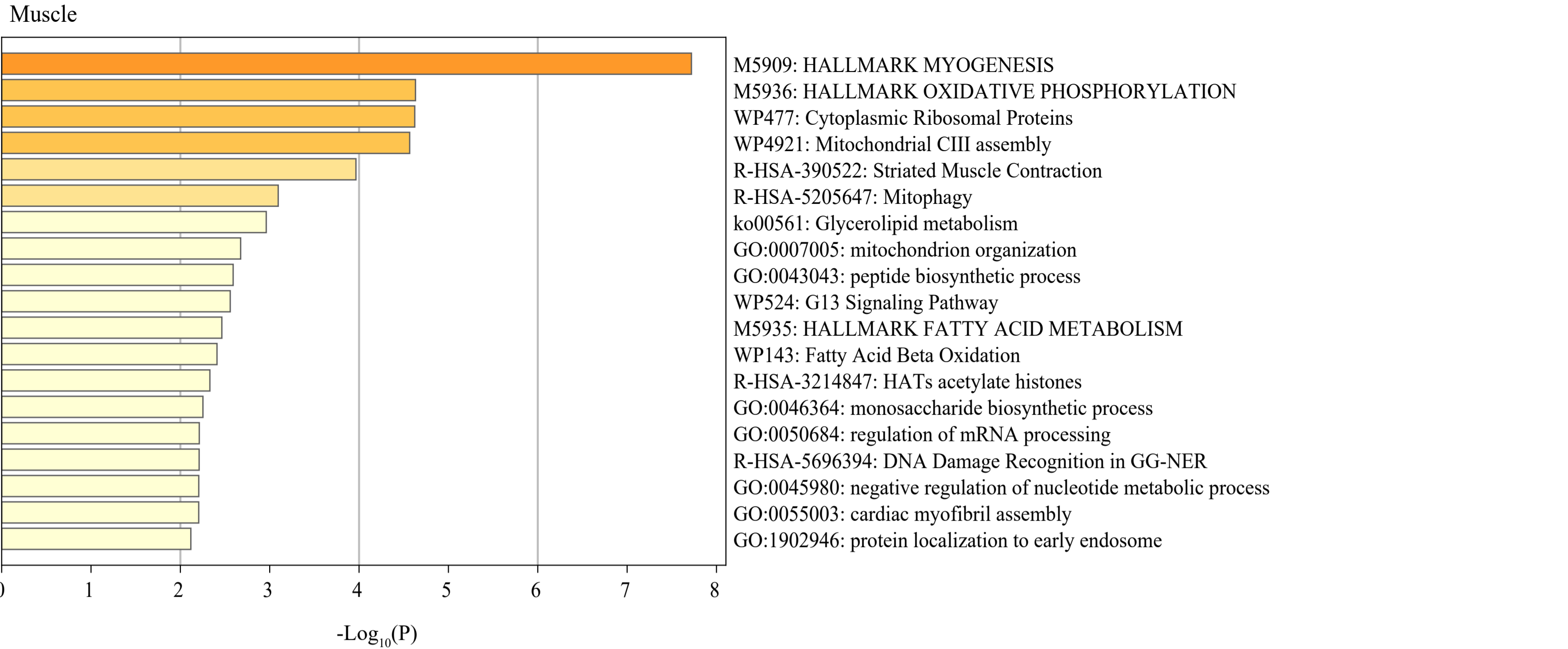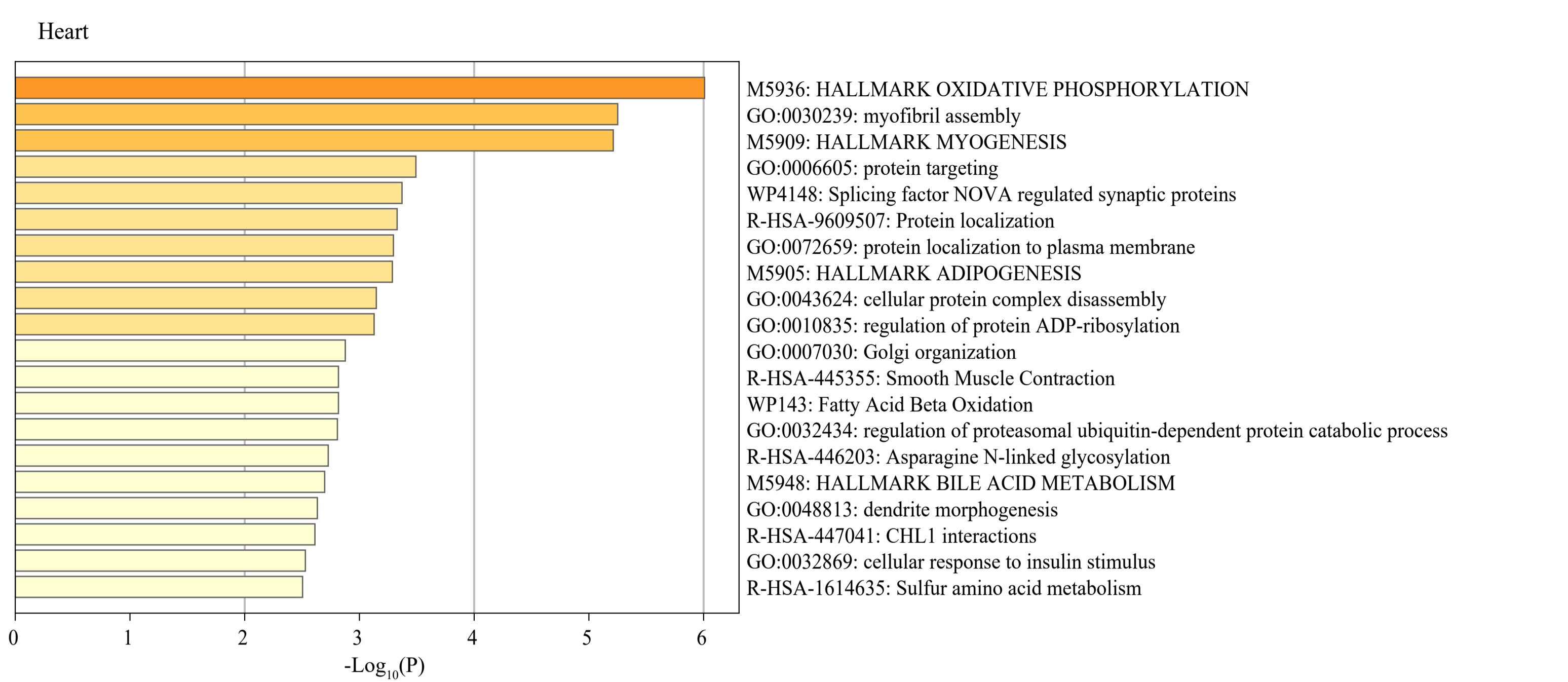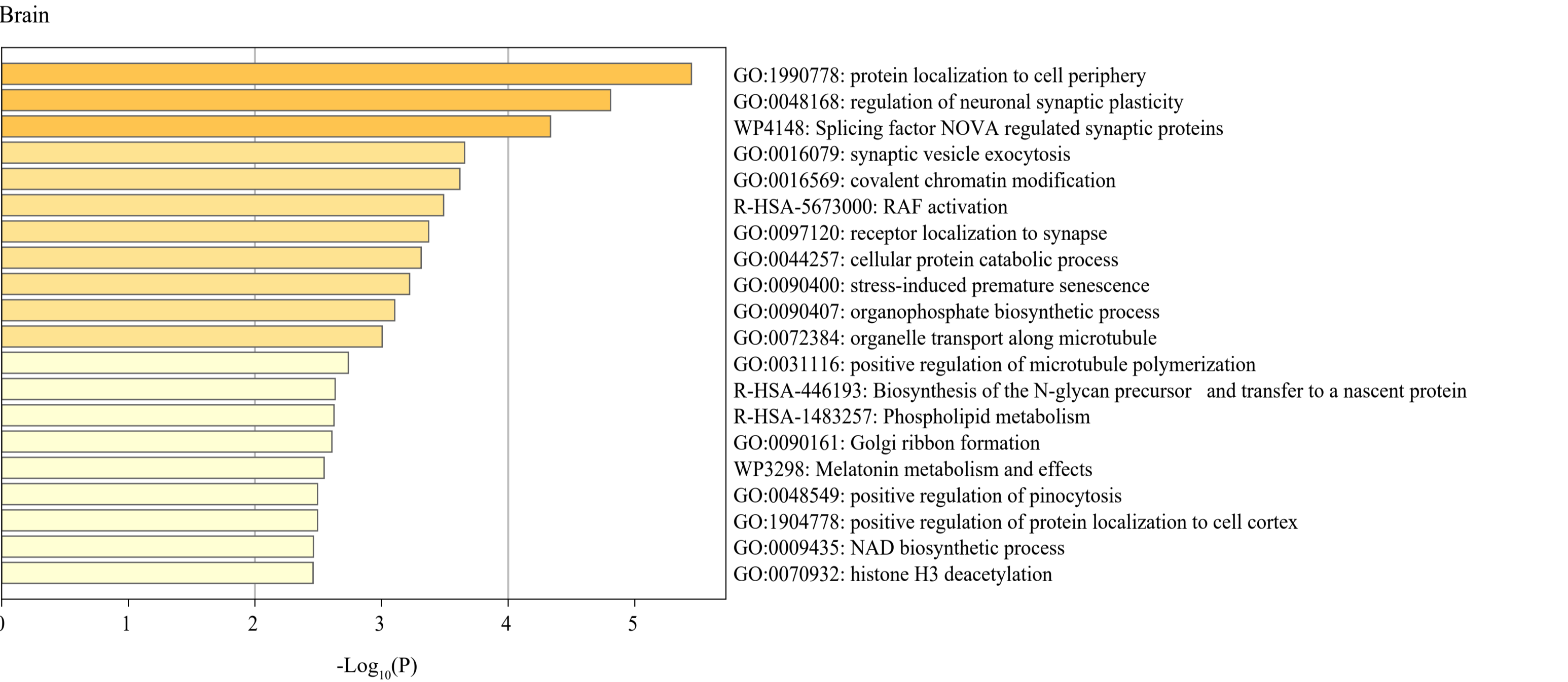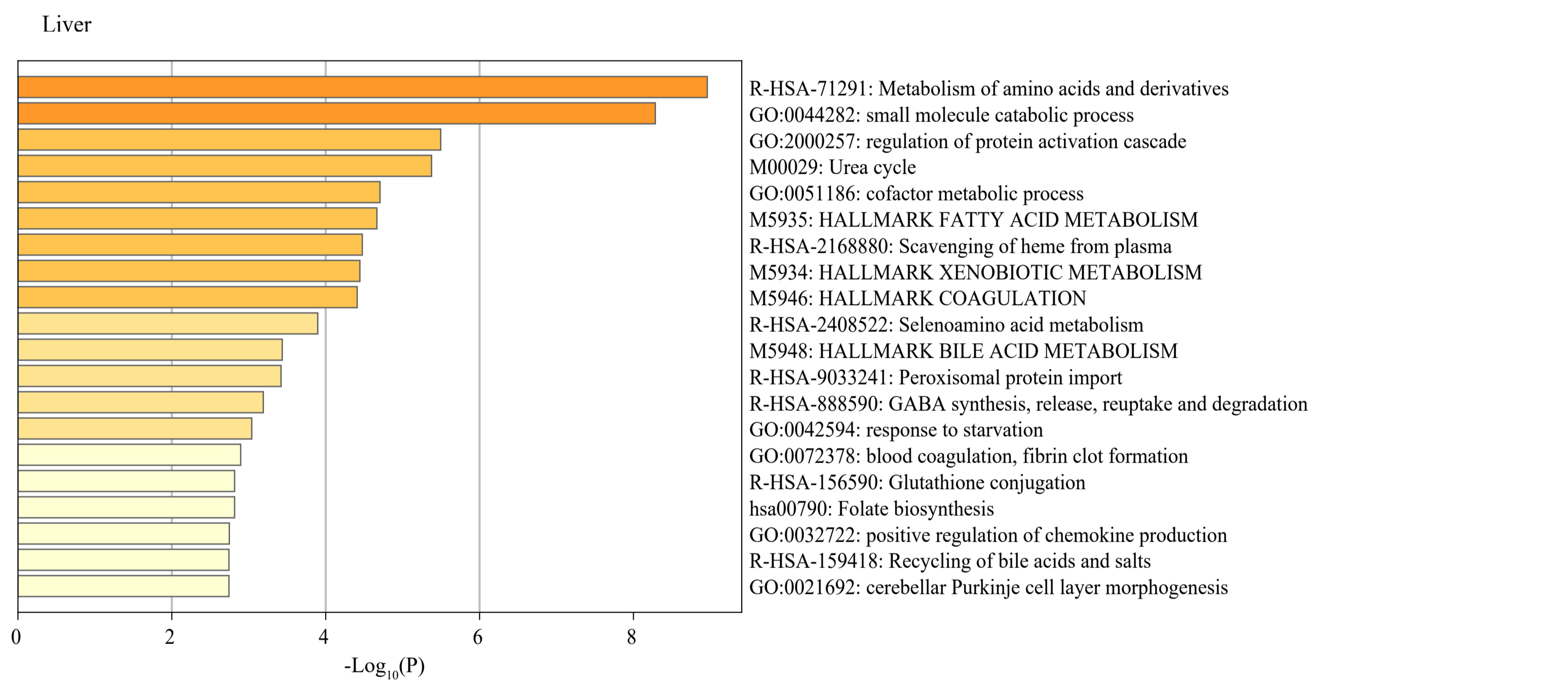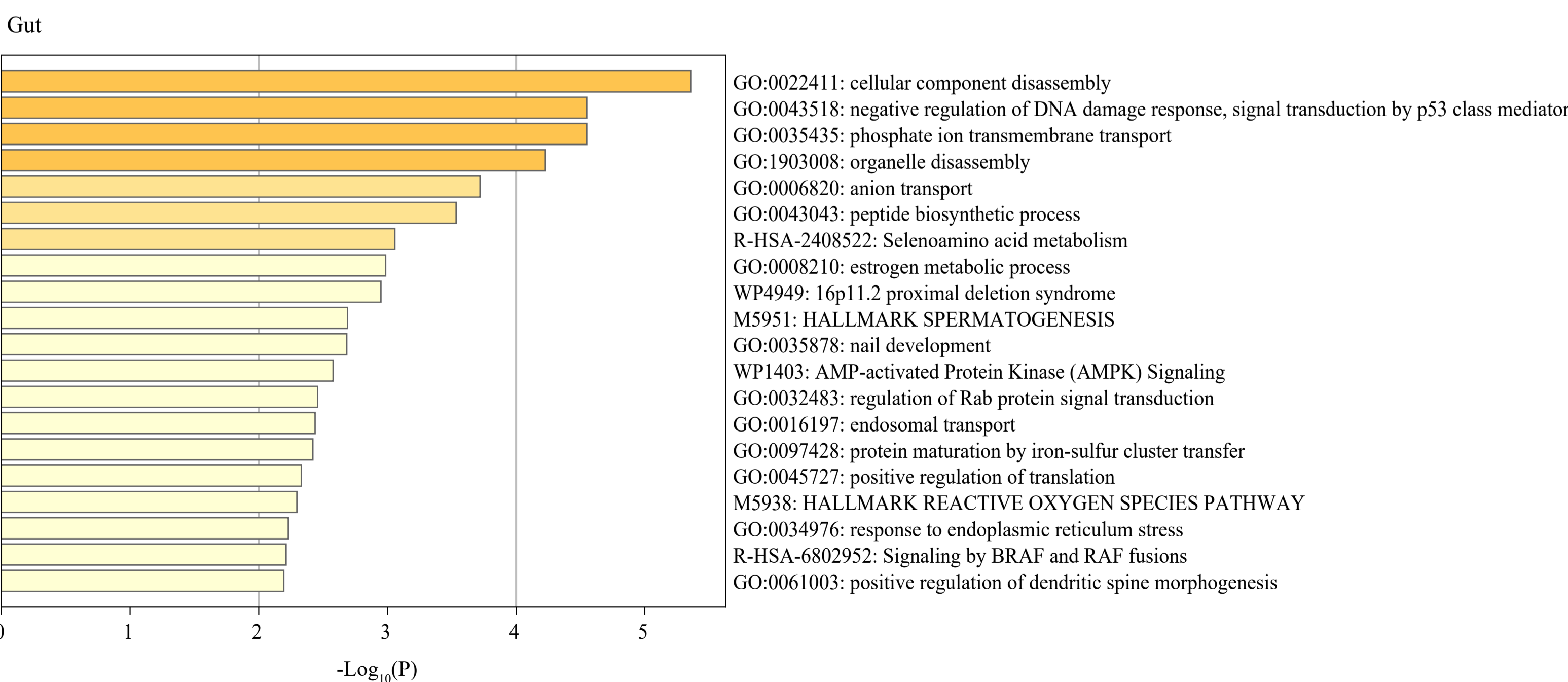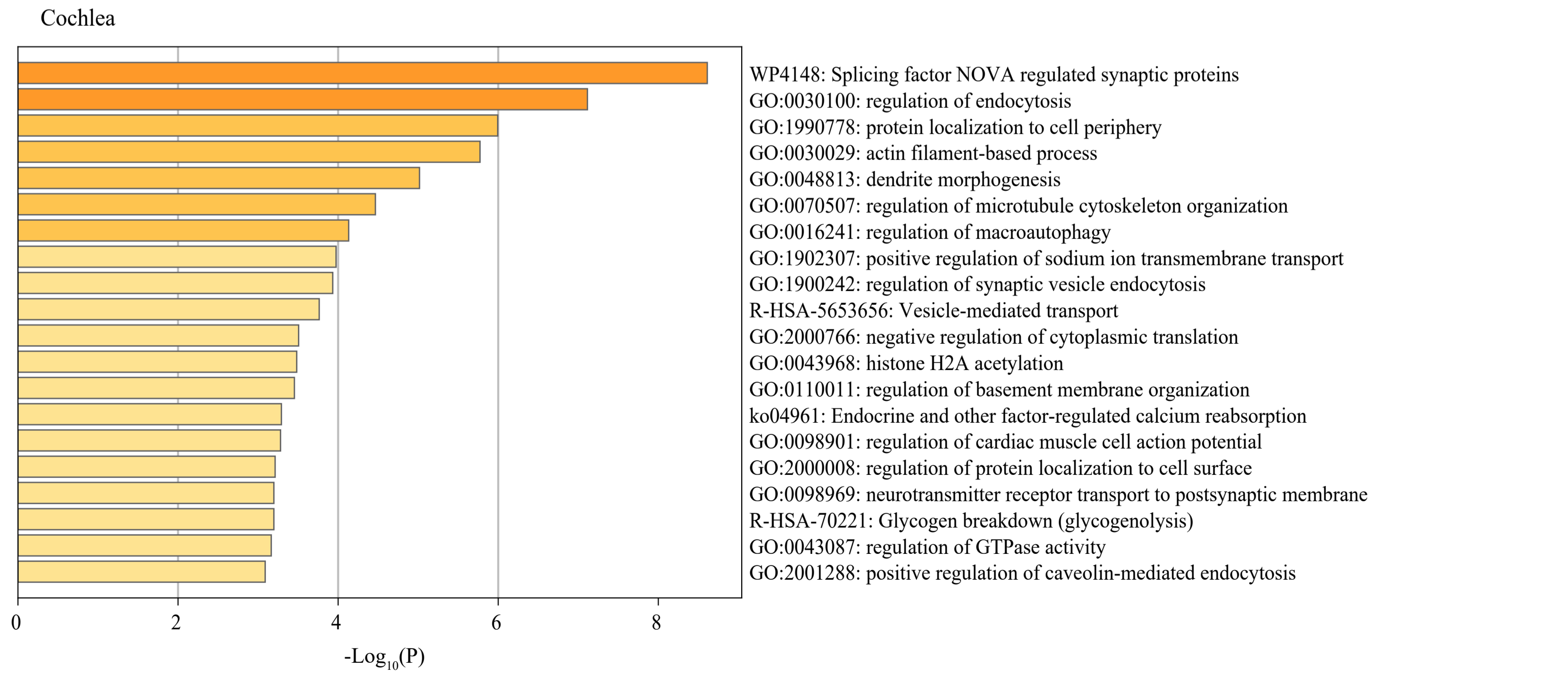

Supplement: Supplementary file 3 — Figure S3 [file ECE3-11-12003-s011.pdf]

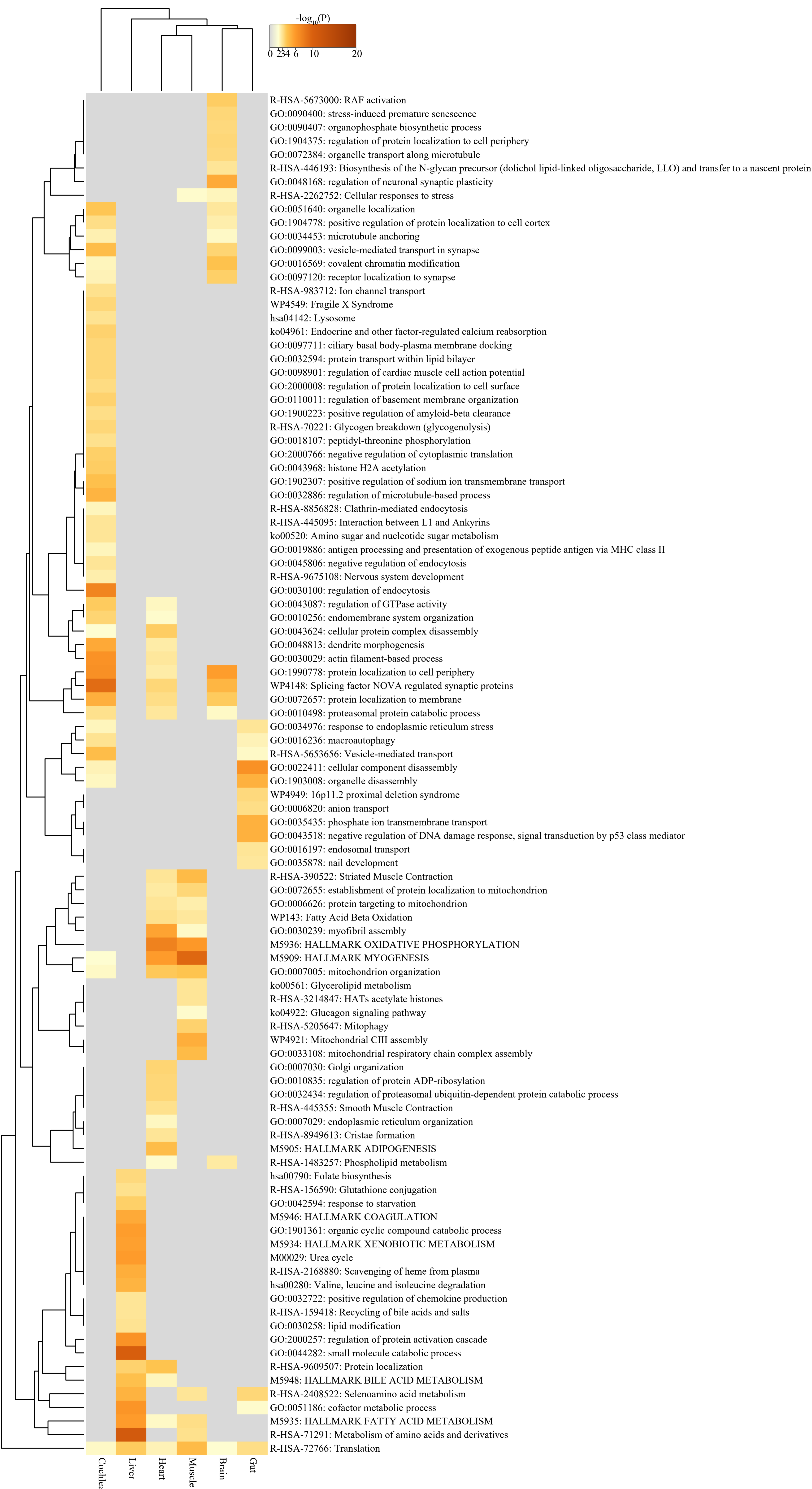

Supplement: Supplementary file 4 — Figure S4 [file ECE3-11-12003-s003.pdf]
